# Forty years on: Uta Frith's contribution to research on autism and dyslexia, 1966–2006

**DOI:** 10.1080/17470210701508665

**Published:** 2007-11-23

**Authors:** Dorothy V. M. Bishop

**Affiliations:** University of Oxford, Oxford, UK

## Abstract

Uta Frith has made a major contribution to our understanding of developmental disorders, especially autism and dyslexia. She has studied the cognitive and neurobiological bases of both disorders and demonstrated distinctive impairments in social cognition and central coherence in autism, and in phonological processing in dyslexia. In this enterprise she has encouraged psychologists to work in a theoretical framework that distinguishes between observed behaviour and the underlying cognitive and neurobiological processes that mediate that behaviour.

## Early academic biography

Imagine a world without computers, printers, pocket calculators, or photocopiers. A world in which the dominant view was that autism was caused by “refrigerator parents”, and the concept of developmental dyslexia was regarded as a self-serving invention of the middle classes. Into such a world came the young Uta Aurnhammer, fresh from the University of Saarbrücken in Germany, where she had been seduced away from her original plan to study art history by the discovery of psychology as an experimental science. Her interest was immediately engaged by the realization that the study of the mind need not rely on mere introspection or dictat, but could be researched using empirical methods, with hypotheses being tested using statistical procedures. Impressed by the spirited attacks by Hans Eysenck (1953, 1957) on psychoanalysis and other nonempirical schools of psychology, Uta decided that the place to go for further training was the Institute of Psychiatry (IOP) in London, and she came to do an internship there in 1964. It was only with hindsight that she became aware of just what a productive mix of influences was based at IOP at that time. Eysenck himself disappointed, being largely inaccessible, but the infant discipline of behaviour therapy was creating excitement, with Jack Rachman, Monty Shapiro, and Reg Beech all looking for applications of this new approach to a wide range of disorders. Uta offered her services as a “work experience” student and soon found herself doing statistical analyses for Reg Beech on a huge clanking calculator, while at the same time trying desperately to improve her English, a task that became a great deal easier when she found herself spending more and more time with Chris Frith. The internship came to an end, and Uta had packed up and sent her luggage off to Germany, but events rapidly took on a life of their own, and she unexpectedly found herself accepted on the clinical psychology course at IOP (then a 13-month diploma), and engaged to Chris Frith. During the clinical course she first encountered cases of “childhood psychosis” (which we would now term “autistic disorder”) and was immediately fascinated at the contrast between the apparently intelligent and attractive appearance of such children and their profound level of handicap. She was convinced, despite the lack of hard evidence and in the teeth of contemporary psychogenic theories, that this must be a disorder with a biological basis, and she started to question the approach of the behaviour therapists, who would try to treat the symptoms, such as gaze avoidance, by conditioning, without any concern for underlying causes. Uta was strongly influenced by the clinical insights of Michael Rutter and Lorna Wing, and she started to hunt out literature that might throw light on the underlying causes of impairment in these strange children. She was drawn to experimental studies by Neil O'Connor and Beate (Ati) Hermelin, which were unique at that time in that they designed studies of developmental disorders from the perspective of experimental psychology. Having selected one of their papers for a journal club (O'Connor & Hermelin, 1959), she was amazed to find that they were based at IOP, in a research unit housed in Nissen huts in the grounds. Despite this insalubrious location, they were an undeniably glamorous pair, more like Hollywood celebrities than psychology researchers, and it took all of Uta's courage to approach them. Uta recounts that her recollection of their meeting was that she was overawed and reverential in their presence, but Ati's subsequent memory was of this challenging young woman who picked their study apart and discussed the flaws with them. Regardless of the reality, Uta clearly impressed and was subsequently offered the chance to study for a doctorate under their supervision, on the topic of “childhood psychosis”. She successfully applied for funding from the Deutscher Akademischer Austausch Dienst and embarked on her studies, only to find, three months into the doctorate, that she did not meet the University of London's eligibility criteria because she did not have a BA. Fortunately the powers that be had the good sense to find a solution by setting Uta a qualifying exam in psychology, and at last she was launched as a research psychologist.

## Autism

In the 1960s, the predominant view of autistic disorder was that its origins were environmental rather than biological. It is all too easy to scoff at such views in the current climate, when genetic and neurobiological accounts of autism are commonplace and widely accepted, but in the light of the evidence they seemed reasonable. For a start, a genetic basis to autism did not seem plausible because the condition did not appear to run in families; there was no evidence of parent-to-child transmission, and it was unusual for more than one child in a family to be affected. Second, there was no indication of any gross neurological damage, and the children looked remarkably normal in physical appearance; unlike many other children of low IQ, their demeanour often suggested high intelligence, giving an impression that there were true abilities locked away beneath the surface. Furthermore, experienced clinicians noted certain deficiencies of social interaction in some parents of children with autism. Taken together, a logical conclusion was that autism was the result of a failure of the child to bond adequately with a parent, leading to a severe disturbance in social interaction. It has to be said that we are still a long way from understanding the neurobiological basis of autism: even with modern imaging techniques it is difficult to detect consistent abnormalities in the brains of children with autism, and the most plausible accounts, in terms of abnormal connectivity and/or deficient neurotransmitters, still lack strong evidence. Nevertheless, what has become clear is that this is a disorder with a genetic basis: The tide started to turn towards acceptance of this idea with a twin study by Folstein and Rutter (1977) and was strengthened by subsequent studies showing an increased incidence of milder “autistic-like” features in relatives of affected children (see Rutter, 2000, for a historical review). Nowadays it is accepted that social oddities do characterize a subset of parents of children with autism, but this is seen as evidence for a shared genetically determined trait in parent and child rather than as an indication of a psychogenic origin to autism. In the 1960s, however, when Uta started to work on autism, only a minority of experts were prepared to countenance the idea that it might be an “organic” condition, and she freely admits that her own conviction that we should look for brain bases rather than family origins was based more on hunch than on evidence. Fortunately, she found herself in an environment where there was sympathy for this hunch, and she was encouraged to do studies of basic perceptual and cognitive processes that might give a clue as to what distinguished these children from others. It is important to realize that autism research in the 1960s and 1970s was a very different enterprise from how it is today. The concept of “high-functioning autism” was not recognized, and the children who were the topic of study had severe cognitive limitations. Some lived in institutions, and others attended the handful of schools that specialized in educating such children, in particular the Sybil Elgar School in West London, which had been opened by the National Autistic Society in 1965. Doing experiments with such children required both stamina and ingenuity, and Uta addressed the difficult problem of devising tasks that would exploit the special interests and cognitive peaks of children with autism. Initially she learned by implementing studies devised by Ati Hermelin: these experiments, included in the classic text *Psychological experiments with austistic children* (Hermelin & O'Connor, 1970) were concerned with the extent to which children's ability to remember verbal or nonverbal material was influenced by meaning. The comparison group were younger children who were matched on memory span: the result, seen for both verbal and nonverbal material, was that meaning improved recall for both groups, but its effect was far stronger in the typically developing controls than in the children with autism. This demonstration of a distinctive cognitive profile strengthened Uta's conviction that we were dealing with a neurological impairment rather than a social inhibition caused by poor parenting. She went on to devise her own experiment, which considered whether children with autism were able to take account of a stress pattern when remembering words. This could be investigated in two ways: first, did children remember more words when a sequence had prosodic structure; second, did they impose a natural prosodic structure on sequences that were presented with no stress? It was found that children with autism took less notice of prosody than did control children (Aurnhammer-Frith, 1969). This led on to work on pattern processing in language and in vision, including an ingenious study of pattern production that may be regarded as the first ever study demonstrating that children with autism had a deficit in generativity (the ability to generate ideas—the inverse of rigidity) (Frith, 1971a).

Throughout her doctoral studies, Uta benefited from the wholehearted support of Ati Hermelin and Neil O'Connor, and from them she learned the skill of designing elegantly simple experiments that illuminated the nature of underlying cognitive deficits. Once her PhD was completed, they offered her a post at their new Medical Research Council (MRC) Developmental Psychology Unit in central London; however, it was clear that she was now expected to develop a new line of research and so, rather than building on the work she had done on autism, she embarked on a series of studies of literacy development. It was only after Neil O'Connor's retirement, when John Morton took over as director of the re-named MRC Cognitive Development Unit in 1982, that Uta returned to studies of autism. Although by this time a more neuropsychological and biological approach to autism was gaining acceptance, in large part due to the influence of Uta's mentors at the IOP, Lorna Wing and Michael Rutter, there was still widespread resistance to the notion that there was a genetic component to the disorder. A remarkable development was the publication of a book entitled *Autistic children: New hope for a cure* by the Nobel laureate Niko Tinbergen and his wife Elisabeth (Tinbergen & Tinbergen, 1983). Tinbergen's reputation was founded on ethological work, and he had the highly original idea that the same observational methods that he had applied to the study of birds could throw light on the problems of children with autism. Unfortunately, his ingenious interpretations of autistic behaviour, and his consequent recommendation of “holding therapy” as a cure, were highly impressionistic and completely lacking in scientific rigour. This therapy involves parents in holding their child for prolonged periods, even if the child is resisting the embrace. The parent tries to establish eye contact and to share feeling with the child throughout the session. Uta wrote a scathing review of the book, but it continued to have a significant impact, with holding therapy continuing to be advocated as a treatment in Continental Europe as well as in the United Kingdom.

There followed a remarkably productive period of research on distinctive cognitive deficits in children with autism, fostered by the unique intellectual atmosphere of the Cognitive Development Unit, where John Morton encouraged his staff to question and debate both theory and experiments. During the early 1980s Uta's research students, Simon Baron-Cohen, Tony Attwood, and Amitta Shah, all made important new discoveries about autism.

Simon Baron-Cohen's influential doctoral studies (Baron-Cohen, Leslie, & Frith, 1985) were stimulated in part by Premack and Woodruff's (1978) work on chimpanzee cognition, in which the term “Theory of Mind” was first used to refer to the cognitive capacity to understand that others may have beliefs, desires, or intentions that differ from one's own. Uta recognized that this had potential relevance for studies of autism, and that Wimmer and Perner's (1983) studies of false belief in children provided an ideal paradigm for investigating Theory of Mind in this population. The basic Sally-Anne task seemed so easy that Uta did not anticipate that children with autism would have any problem in responding correctly, and she was amazed when this task proved such a sensitive tool for revealing the underlying deficits in social cognition. Alan Leslie joined the collaboration working on this topic, providing a theoretical viewpoint that regarded Theory of Mind as a subset of more general metarepresentational knowledge. Throughout this period, the group benefited from John Morton's insistence that it was not enough just to describe what had been found: it was important to place it in a theoretical context that would allow for the development of new predictions that could be tested experimentally. It is not exaggerating to say that the Theory of Mind work transformed the conceptualization of autism by psychologists. Prior to this work, psychological studies on autism were not very coherent. The earlier studies on perceptual processes and pattern perception by Frith and others were clearly important, but it was difficult to relate these findings to the symptoms of the disorder, especially the core social impairments. In the United Kingdom, much theoretical debate had focused on the question of whether autism could be explained as a language disorder (e.g., Churchill, 1978), and new insights had been gained by explicit comparisons of children with autism and those with receptive language disorders (Bartak, Rutter, & Cox, 1975), but the core features of social impairment remained unexplained. In the United States, Marian Sigman, Peter Mundy and their collaborators were doing detailed observational studies of social interaction in children with autism, noting deficiencies in shared attention and nonverbal communication (Sigman, Mundy, Sherman, & Ungerer, 1986). The Theory of Mind work provided a way of conceptualizing social impairment as stemming from a cognitive failure in a system that computes representations of other minds and their contents. It not only made theoretical sense; it also provided new avenues for thinking about intervention, and many people working in special education found it helped them to understand why a child with autism might react in an unusual way to social situations. The fact that Theory of Mind could be assessed using a relatively simple experimental task must also have played a part in the enormous volume of research stimulated by the 1985 paper. This included fruitful collaborative studies with Josef Perner, who had hitherto worked solely on typical development, but who continued to develop new ways of looking at Theory of Mind, including the now-famous Smarties task (Perner, Frith, Leslie, & Leekam, 1989).

Tony Attwood was a part-time graduate student who also worked as a clinical psychologist. Previous studies had suggested that children with autism made deficient use of gestures: Attwood's studies showed that this was an oversimplification, and that the function of gestures was key. His findings related neatly to the Theory of Mind theorizing, showing that instrumental gestures, which do not require any interpersonal understanding, were intact, whereas expressive gestures, which serve a purely communicative function, were never observed in children with autism (Attwood, Frith, & Hermelin, 1988).

Amitta Shah's work was distinctively different. She focused on the unusually good performance on embedded figures and block design tasks in children with autism (Shah & Frith, 1983, 1993). Interest in strengths as well as weaknesses in the cognitive profile of autism was a distinctive aspect of work at the Cognitive Development Unit, and was key to the development of the theory of weak central coherence.

In parallel with these exciting developments on the cognitive front, there was a gradual broadening of the concept of autism. An epidemiological study by Lorna Wing and Judith Gould had already drawn attention to the existence of a large number of children with social abnormalities who did not have classic Kanner syndrome but nevertheless shared many features with these cases (Wing & Gould, 1979). A subsequent report noted that although most children with these characteristics were mentally retarded, this was not true for all (Wing, 1981a). At the same time, Asperger's report of a syndrome akin to autism but accompanied by relatively good language skills was summarized in English (Wing, 1981b), starting a lively debate as to whether this should be regarded as a subtype of autism or a distinct disorder. Meanwhile, as described above, genetic and family studies by Michael Rutter and associates were forcing researchers to the conclusion that there was a spectrum of autism, ranging from classic Kanner syndrome at one extreme to a broader phenotype at the other extreme, where the affected individual might function normally in society but have mild abnormalities of social interaction or communication. This was the start of a change in the nature of research on autism. Hitherto, cognitive studies had focused on children with intellectual retardation; gradually, as the concept of autism broadened, researchers turned more and more to the so-called high-functioning cases, who were far easier to work with and who, it was hoped, might throw autism-specific deficits into much sharper focus. Uta translated Asperger's text in 1991, and subsequently she became one of the world's experts in this syndrome.

One graduate student who took advantage of the broadened concept of autism to study cases of high-functioning autism and Asperger syndrome was Francesca Happé, who arrived at the Cognitive Development Unit in the late 1980s. Language in autism had always interested Uta, and she could see that investigation of the relationship between language and Theory of Mind would be a fruitful topic for study. Happé (1991) used Sperber and Wilson's (1986) Relevance Theory to generate predictions about how children with autism would interpret non-literal language and found an impressive fit between theory and data. For Uta, the puzzle of language in autism made sense if one recognized that the core deficits were pragmatic failures in the appreciation of relevance, with other aspects of language impairment (e.g. in structural aspects such as grammar and phonology) being correlates of the condition rather than key features (Frith, 1989b).

In 1989 *Autism: Explaining the enigma*, Uta's synthesis of more than two decades of work on cognitive bases of this disorder (Frith, 1989a), appeared. She made a strong case for autism as a neurobiological disorder and presented compelling evidence for Theory of Mind as a core area of deficit, but she also drew attention to the a new idea concerning Weak Central Coherence as another aspect of the autistic mind. Unusually for an academic text, this was written in a clear, informal, and engaging style and accompanied by charming illustrations, and it immediately became a best-seller, with translations into numerous other languages.

Francesca Happé stayed on at the Cognitive Development Unit after completing her doctorate, and she and Uta worked together to consider how Weak Central Coherence related to other aspects of autistic cognition, including strengths in certain aspects of memory and perception as well as deficits in the ability to use context. This culminated in a detailed exposition of the Weak Central Coherence account by Frith and Happé in *Cognition* in 1994 (see also Happé & Frith, 2006, for an update of the theory).

The mid 1990s saw another major development that was to fundamentally change the course of Uta's research: the development of functional brain imaging. The earliest studies were done using the positon-emission tomography (PET) system at the MRC Cyclotron Unit at the Hammersmith Hospital, where Chris Frith was involved in developing activation paradigms using this new technology. Initially PET was used to probe the neural basis of Theory of Mind in normal adults (Fletcher et al., 1995); subsequently the same paradigm was applied to adults with Asperger syndrome, who showed a specific decrease of activation in the medial prefrontal cortex, which was the region activated by Theory of Mind tasks in control participants (Happé et al., 1996). Work on brain imaging gathered pace with the development of imaging facilities in central London at the Wellcome Department of Cognitive Neurology, where Chris Frith and his colleagues pioneered methods for analyzing brain activation in both PET and functional magnetic resonance imaging (fMRI) paradigms. Studies using fMRI provided yet more evidence for involvement of the medial frontal lobes in Theory of Mind (Gallagher, Frith, & Snowling, 2000).

Chris Frith has always been a major influence in Uta's academic life, right from the earliest days at the IOP, when he helped her through her struggles with English language and culture. During 1970s and 1980s Uta and Chris would always discuss ideas and read one another's papers, but it was in the 1990s that their research collaboration really took off, as the new imaging techniques became increasingly important. In more recent years, their work has moved in a new and fruitful direction, with the integration of neuropsychological work on autism and schizophrenia. Both disorders involve abnormalities in social cognition as well as executive function impairments, but whereas people with autism under-utilize Theory of Mind, it appears that Theory of Mind is overactive in those with para-noid–schizophrenic symptoms, who see meaning and communication in situations where it is not intended. The cross-fertilization between research on these two disorders has led to innovative theoretical approaches to both autism and schizophrenia, engendering a line of work that promises to be enormously productive over the next few years.

## Dyslexia

Uta's interest in causes of variation in children's reading goes right back to her undergraduate time in Saarbrücken, when she did a project on visuoperceptual skills and literacy development. Marianne Frostig's ideas on perceptuo-motor causes of learning disabilities were influential at the time (see: http://www.frostig-gesellschaft.de/M_Fro_EN.htm), and Uta's project involved training children to draw a line between two guidelines that got increasingly close together, to see how this related to literacy. The challenge of such a project in the pre-photocopier era should not be underestimated: To generate response forms, Uta needed to use a stylus to scratch out a template onto a special wax-impregnated paper, which could then be put through a special rotary printing press. She used this not only to make simple lines, but also drew pictures to create a “storyline” for what was otherwise a very boring task. The results were not conclusive, but Uta was interested to note that some children reversed letters, reading “b” as “d” and vice versa. She followed up this observation in her dissertation at IOP and subsequently picked it up again in her earliest papers on reading in the early 1970s (Frith, 1971b, 1974).

During the 1970s, three things happened that led Uta to radically rethink her views on dyslexia. First, during the summer of 1974, she made her first foray to the United States, spending a month at the University of Delaware, participating in an Institute on Reading and Child Development, sponsored by the Society for Research in Child Development. Around 30 post-grads and junior scientists took part, all living in a dorm on campus, and every few days new lecturers—including Lila Gleitman, Isabelle Liberman and Donald Shankweiler—would descend to give an intensive introduction to their own research. This experience led Uta to shift from regarding dyslexia as a disorder of visual perception to appreciate the important role of linguistic, especially phonological, processing.

The second factor was Uta's discovery of the extent to which reading and spelling could be dissociated in dyslexia. Particularly in older and brighter individuals, one could find cases where spelling problems persisted in a person who could read adequately. Uta became intrigued by this imbalance in skills, which led her to reflect on the different types of cognitive process involved in word recognition and written production. This led to her editing an influential book on this topic (Frith, 1980). This was very different from previous educational texts on spelling, because it brought together perspectives from cognitive neuropsychology, linguistics, and developmental psychology. The cognitive neuropsychology perspective was only just beginning to be applied to children, with the recognition that by studying cognitive development in atypical populations one could throw light on normal developmental processes. Chapters by Uta's colleagues Rick Cromer and Barbara Dodd, on spelling in language-impaired and hearing-impaired children, respectively, exemplified this approach.

The third major influence on Uta's thinking was Maggie Snowling, who started her graduate studies at the Cognitive Development Unit in 1976. Maggie already had an intense interest in dyslexia, a problem that affected members of her immediate family, and she sought out Bevé Hornsby, who had founded the Hornsby International Dyslexia Centre in London and was developing pioneering new approaches to intervention (Hornsby & Miles, 1980). This gave Maggie experience of assessing and teaching children with severe and selective reading difficulties, who provided ample evidence of the importance of phonological processing problems in leading to literacy impairments. Her thesis studies built on the foundations set by the original Hermelin and O'Connor work: carefully designed small-scale studies in which performance of a disordered group was compared with that of younger children matched on performance on a key measure—in this case, reading level. This led to one of the earliest demonstrations that dyslexic children were poor even relative to reading-age matched controls on tasks involving phonological processing, even when no written language was involved (Snowling, 1981). Subsequently, Uta and Maggie carried out a study explicitly comparing reading skills in autism and dyslexia, showing that whereas processing of meaning was disrupted in the former group, processing of sounds was impaired in the latter (Frith & Snowling, 1983).

One of the things that made John Morton a remarkable director of the Cognitive Development Unit was the fact that he did not start out as a developmental psychologist: his background was in mainstream experimental psychology. The influence of this background, with its insistence on articulation of a clear theoretical framework, had a marked impact on his colleagues and students at the Unit. It also meant that John continued to move easily between the worlds of adult and child psychology, and encouraged interactions between the two domains. Thus it came to pass that Uta found herself participating in a meeting on Surface Dyslexia, a topic of considerable interest to neuropsychologists working on acquired dyslexias, which generated a book in which she was encouraged to lay out a theoretical position specifying how developmental dyslexia related to normal stages of reading development (Frith, 1985). This contrasted with models of reading development adapted from adult neuropsychology that attempted to describe possible processes causing change in behaviour. The model led to increasing international recognition of her work on reading, with an invitation to participate in a meeting of the prestigious Orton Society (Frith, 1986).

For the next few years, work on dyslexia took a back seat, as Uta again turned to concentrate on autism, where such remarkable progress was being made. However, the advent of brain imaging led to an opportunity to work on one of the first PET studies of dyslexia, in collaboration with Eraldo Paulesu, an Italian neurologist who was working at the Wellcome Department of Cognitive Neurology with Chris Frith. The crucial insight that stimulated these studies was that dyslexia was not just a childhood disorder: it persisted into adulthood, with residual signs being detectable even in those who appeared to have compensated for their difficulties. Maggie Snowling's long-term follow-ups of cases she had seen in childhood emphasized this point, and with her help it was possible to recruit adults who had been studied as dyslexic children. They showed a distinctive pattern of brain activation during phonological tasks, in which the normal connectivity between posterior and anterior areas appeared to be abolished (Paulesu et al., 1996).

Two other lines of work were started in the late 1990s: high-risk and crosslinguistic studies. The high-risk study, done in collaboration with Alison Gallagher and Maggie Snowling, capitalized on the evidence that dyslexia was a strongly genetic disorder, by selecting 3-year-olds whose parents had dyslexia, so that they could be studied before they were introduced to reading. Literacy impairment at 6 years was substantially more frequent in the at-risk than the control group and was associated with early language deficits, confirming the view that the problems of children with dyslexia encompass oral as well as written language (Gallagher, Frith, & Snowling, 2000). Maggie Snowling tells us more about the later phases of this study in her chapter in this volume (Snowling, 2008).

Cross-linguistic studies provide a particularly rich testbed for theories of reading disability, because they enable one to test predictions about the manifestations of dyslexia in languages that have different relationships between orthography and phonology. One set of studies arose from Uta's collaborations with Heinz Wimmer. Originally, they had worked together on Theory of Mind, but Wimmer was looking for something new, and comparisons of reading development in German and English was a topic that had not previously been adequately addressed. There followed a series of studies by Wimmer and his colleague Karin Landerl that emphasized the dangers of relying solely on one language, English, when developing a theory of dyslexia. Although there were many similarities between profiles of dyslexia in the two languages (Landerl, Wimmer, & Frith, 1997), the German-speaking children also posed problems for the theory that dyslexia was primarily a disorder of phoneme awareness (Landerl & Wimmer, 2000).

The other opportunity for cross-linguistic study arose from the collaboration with Eraldo Paulesu. Uta was able to obtain EU funds for a PET study of dyslexia in English, French, and Italian, three languages that contrasted considerably in the regularity of their orthography. This showed that, despite different levels of behavioural impairment, reduced activity in the same region of the left hemisphere was seen in dyslexics from all three countries. As the title of the paper aptly put it, there appeared to be a biological unity underlying the cultural diversity due to different orthographies (Paulesu et al., 2001). These same participants continue to be studied using new methods of imaging, with a recent voxel-based morphometry, finding altered density of grey and white matter of specific left hemisphere regions, and altered connectivity between regions (Silani et al., 2005).

The work on dyslexia nicely illustrates how Uta has succeeded in integrating different levels of explanation—neurobiological, cognitive, and behavioural—in her model of this disorder. Her theoretical approach has sharpened our thinking about causal pathways, and to recognize that we will only gain a full understanding if we take a cognitive neurobiological perspective (Frith, 1997).

## Conclusion

Uta describes herself as someone whose early academic career was characterized by enormous good fortune: first, the fact that, by chance, she found herself at one of the few universities in Germany that taught experimental psychology and provided her with the opportunity to attend lectures in a subject far removed from her major discipline; second, her arrival at the IOP at a time when it was alive with iconoclastic young scientists who favoured empiricism and neurobiology over traditional psychoanalytic approaches; third, the chance drop-out of a potential student that allowed her to obtain a diploma in clinical psychology; and, fourth, the sequence of events that led her to be taken on as a PhD student by Hermelin and O'Connor, whose unique experimental approach was combined with a remarkable generosity of spirit towards their new student. The marital and academic partnership with Chris Frith has been the strongest formative influence on Uta's career and would never have happened had she not crept away from her art history lectures to find out what this psychology stuff was all about. It is intriguing to wonder what Uta would be doing now had any one of these events not occurred. One thing that is certain, however, is that Uta's success is not due simply to serendipity. She benefited from the opportunities provided because she matched them with her keen scientific interest and a talent for social communication that made her an excellent student, supervisor, and collaborator. For Uta, a theory was an important step in the process of hypothesis formation, and the goal was to test it rigorously rather than to shore it up at all costs. Her talent for devising simple, child-friendly experiments that cut to the heart of a question remains unsurpassed. Uta was indeed fortunate to have regular contact with so many talented and original academic influences, but she must also be credited for being someone who could extract the maximum from these, being ready to listen, debate, and learn wherever new ideas were being discussed. As well as the many distinguished academics whom she credits with influencing her work, she also pays tribute to the parents of children with autism and dyslexia, whose insights into their children's cognition have provided a rich source of ideas.

This chapter has barely been able to scratch the surface concerning Uta's own work and the formative influences on it. In the remainder of this book we will hear from her students and collaborators, who will amply demonstrate the important influence she has had on subsequent generations of researchers.
